# Development of the Leiden Independence Questionnaire for Support Staff: a measure of staff behaviour regarding promoting independence of people with intellectual disabilities

**DOI:** 10.1111/jir.12574

**Published:** 2018-12-05

**Authors:** J. Sandjojo, W. A. Gebhardt, A. M. E. E. Zedlitz, J. Hoekman, E. Dusseldorp, J. A. den Haan, A. W. M. Evers

**Affiliations:** ^1^ Institute of Psychology, Health, Medical and Neuropsychology Unit Leiden University Leiden The Netherlands; ^2^ Leiden Institute for Brain and Cognition (LIBC) Leiden University Leiden The Netherlands; ^3^ Raamwerk Noordwijkerhout The Netherlands; ^4^ Institute of Education and Child Studies, Clinical Child and Adolescent Studies Leiden University Leiden The Netherlands; ^5^ Institute of Psychology, Methodology and Statistics Unit Leiden University Leiden The Netherlands; ^6^ Department of Psychiatry Leiden University Medical Center Leiden The Netherlands

**Keywords:** independence, intellectual disabilities, questionnaire, self‐management, support staff

## Abstract

**Background:**

Support staff of adults with intellectual disability (ID) play an important role in promoting independence in home and community settings. However, little is known about the types of behaviours staff should use to promote independence and instruments that assess such behaviour do not yet exist. The aim of this study was therefore to develop and initially validate a reliable questionnaire that measures the degree to which support staff display behaviours that promote independence in people with ID.

**Method:**

The Leiden Independence Questionnaire for Support Staff (LIQSS) was constructed to measure the extent to which support staff promote independence in people with ID. The LIQSS was completed by 142 staff members working with people with ID. For the psychometric evaluation of the LIQSS, a principal component analysis was performed with an oblique rotation in all items. Next, the principal component analysis was performed with a forced three‐component extraction, and three sub‐scales were computed. To assess internal consistency, Cronbach's *α* was calculated for each of the sub‐scales.

**Results:**

The LIQSS was found to consist of three internally consistent (Cronbach's *α* was respectively 0.92, 0.79 and 0.76) and meaningful components: (1) communication, agreements and coordination; (2) positive encouragement and tailoring; and (3) supporting independent performance. The final 22 items had factor loadings between 0.44 and 0.91 on their corresponding component and a minimal difference in loading to the other factors of 0.20.

**Conclusions:**

The LIQSS appears to be an instrument with positive face validity and reliability (internal consistency) that assesses the degree to which support staff promote independence in people with ID. To increase the instrument's value for both scientific research and clinical practice, studies should focus on the further validation of the LIQSS.

## Background

There is international consensus that people with intellectual disability (ID) should live as independently as possible (United Nations 2006). However, as our Western society is becoming more complex, being independent has become increasingly challenging for people with ID, leading to a growing demand for care (Netherlands Institute for Social Research 2014). People with ID often struggle with managing their personal care, household, community or work activities (Laarhoven and Van Laarhoven‐Myers 2006; Dusseljee *et al*. 2011; Smith *et al*. 2015) and therefore are often dependent on others for support (Schalock 2004; Hale *et al*. 2011; Spriggs *et al*. 2017; Vilaseca *et al*. 2017). Being independent, however, is important for people with ID (Kuijken *et al*. 2016) and has been related to greater happiness, satisfaction (Bond and Hurst 2010; Haigh *et al*. 2013) and quality of life (Sigafoos *et al*. 2005; Dollar *et al*. 2012). Therefore, to enhance the quality of life of people with ID, it is important to promote their overall self‐management and independence in particular. Support staff could play an important role in this regard. However, clear indications are lacking on what type of staff behaviours promote independence in people with ID and valid and reliable instruments that target these behaviours are missing.

Independence, just as self‐reliance, can be defined as the ability to take action to manage one's affairs and to provide for oneself, thereby solely relying on one's own efforts, resources, judgement and abilities, without requiring help or support from others (Sandjojo *et al*. 2018). Someone's level of independence can be placed on a continuum, with complete dependence at one extreme and complete independence at the other (Aldridge 2010). Although no one is completely independent in all areas, the goal for people with ID is to be as independent as possible. The concept of independence is part of the overarching term ‘self‐management’. Self‐management has been defined and described in various ways (Browder and Shapiro 1985; Harchik *et al*. 1992; Ferretti *et al*. 1993). Taking these studies together, self‐management can be defined as the set of actions and cognitions that a person deliberately undertakes to change or maintain his or her behaviour in order to achieve self‐selected outcomes. As self‐management thus involves the capacity to manage one's behaviour, it includes the concept of independence. Additionally, related terms include self‐determination and autonomy, which are centred on making self‐selected choices. Self‐determination and autonomy are separate constructs that both concern acting as the primary causal agent in one's life, thereby having personal control over making choices and decisions in order to lead one's life according to one's own preferences, free from external influences (e.g. Wehmeyer *et al*. 1996; Shogren *et al*. 2015; Sexton *et al*. 2016).

Traditionally, within residential care settings for people with ID, there may be less emphasis on promoting aspects of self‐management (Felce *et al*. 2000). A way to improve self‐management in people with ID could be through focusing on support staff to help promote independence. Changing the way support staff behave can have a positive impact on client outcomes (Hastings 2010). For example, if staff encourage clients to handle things themselves, instead of taking over from them, this could increase clients' independence and reduce passivity and ‘learned helplessness’ (Sigafoos *et al*. 2005). Although there are only a few studies that directly targeted staff behaviour with regard to promoting self‐management in this population (e.g. Wong and Wong 2008; Sandjojo *et al*. 2018), staff often play a role in self‐management interventions that target people with ID directly. In a recent systematic literature review on interventions that aimed to promote self‐management in daily life of people with ID (Sandjojo *et al*. in preparation), it was found that it was always the provider of the intervention (e.g. support staff member) who applied behavioural change techniques to promote self‐management. Mostly a combination of techniques was used, such as modelling, instructing, prompting and providing feedback, which all seemed to be effective. However, when it comes to promoting independence in particular in people with ID, no studies have been conducted to date on the specific types of staff behaviours that are necessary.

Furthermore, instruments that assess independence‐promoting behaviour of staff do not yet exist. In the few previous studies that focused on staff behaviour in relation to promoting self‐management in people with ID, various types of measures were used. For example, in one study, an observer made detailed records of staff's skills and behaviours while they were working (e.g. interaction with clients, providing opportunities for involvement and choice making) (Beadle‐Brown *et al*. 2012). Other studies conducted interviews with trained staff to evaluate how a training affected their behaviour (Totsika *et al*. 2008), attitude, knowledge and skills (Sandjojo *et al*. 2018). Wong and Wong (2008) constructed their own scale to assess staff's attitude, knowledge and skills, with a specific focus on facilitating self‐determination of people with ID. Their instrument mostly contained statements, but they also used vignettes to assess staff's responses. Based on these four studies, it seems that it is generally important that support staff closely involve people with ID in activities and decision‐making. However, it is not yet clear which staff behaviours are specifically important for promoting independence in people with ID, as this was not specifically addressed in these studies. Furthermore, there are concerns about the reliability and validity of measures that evaluate staff behaviour and the effect of staff trainings, especially measures that can be easily completed. This implies the need for the development of validated measures to assess staff practice (Rose *et al*. 2012).

In the current study, we developed and initially validated a reliable questionnaire that assesses the degree to which support staff promote independence in people with ID.

## Methods

### Participants

For the development of the LIQSS, we approached 174 support staff members of Raamwerk and 's Heeren Loo who worked in residential homes or day‐care services with adults with moderate to mild ID without significant physical impairments. There were 142 staff members who participated: 109 from Raamwerk and 33 from 's Heeren Loo. The response rate was 81.6%. The descriptive statistics of the whole sample are displayed in Table 1. In general, there were more female participants than male participants, and most participants completed intermediate secondary vocational education and training. The majority of the participants worked as a regular staff member, as opposed to working as a personal tutor, and most participants worked within a residential setting.

**Table 1:** Descriptive statistics of participating staff members

	Whole sample (*n* = 142)
Gender, *n* (%)	
Male	49 (34.5)
Female	93 (65.5)
Age in years, M (SD)	37.9 (12.5)
Level of education, *n* (%)	
Lower secondary vocational	3 (2.1)
Intermediate secondary vocational	89 (62.7)
Higher professional	31 (21.8)
Unknown	19 (13.4)
Work experience in years, M (SD)	13.4 (9.5)
Work setting, *n* (%)	
Homes	104 (73.2)
Day‐care services	38 (26.8)
Role, *n* (%)	
Personal tutor	42 (29.6)
Regular staff	97 (68.3)
Unknown	3 (2.1)

Independent samples *t*‐tests were conducted for numerical variables and chi‐squared tests for categorical variables.

M, mean; SD, standard deviation.

### Instrumentation

To construct our questionnaire, the Leiden Independence Questionnaire for Support Staff (LIQSS), we based our method on the intervention mapping approach by Bartholomew *et al*. (2011), who devised a protocol for developing effective behaviour change interventions. One of the intervention mapping steps concerns making an overview of performance objectives, which in our case concerned staff behaviours that are important when wanting to promote independence in people with ID. This was performed by having discussions with the first author and members from our expert group, in which we brainstormed about all the behaviours that support staff should display to promote independence in people with ID. Based on these discussions, the list of performance objectives was revised and complemented several times until consensus and saturation was reached. Based on this list, we clustered the performance objectives into several domains (i.e. setting goals, motivating people with ID, supporting the learning process and coordinating with others). The list was then converted into a questionnaire, in which no explicit reference was made to these domains. For example, the performance objective ‘Support staff give clients room to make mistakes’ was converted into the questionnaire item ‘I give clients room to make mistakes’. The final version of the questionnaire that was used for the psychometric evaluation contained 32 items reflecting the extent to which staff expressed behaviours that promote independence (Appendix A1). Participants were instructed to carefully read the items and to indicate to what extent these items were applicable to them in the past 2 weeks on a scale from 1 (*not at all/never*) to 7 (*completely/always*). By having our group of experts review the appropriateness, relevance and completeness of the final scale, we could ensure its face validity.

### Procedure

The study protocol was evaluated by the Medical Ethics Committee of the Leiden University Medical Center. It was declared that neither formal medical ethical approval nor written informed consent was required and that there were no objections to conducting the study. The LIQSS was developed by a group of experts (*n* = 12), including researchers, psychologists, and support staff and managers of two care organisations (i.e. Raamwerk and the Academy of Independence). Their years of work experience in the field of ID ranged from 2 to 22 years. The first version on the questionnaire was piloted with four support staff members in a think‐aloud study (French *et al*. 2007). While filling in the questionnaire, staff expressed all their thoughts out loud, and afterwards, they were asked how they evaluated the questionnaire (e.g. whether anything was unclear, whether it fit our purpose and whether it addressed all of the relevant behaviours). Based on the results of this think‐aloud study, some adaptations were made such as clarifying an item with an example, rephrasing three items and slightly adapting the instructions. Data collection was carried out in collaboration with the organisations Raamwerk and 's Heeren Loo, both care organisations for people with ID in the Netherlands. All participating staff were informed about the study beforehand by e‐mail or by the psychologist or remedial educationalist of their team. All staff members were asked to participate on a voluntarily basis, which they could do during work hours.

### Data analyses and statistics

The data were analysed with (IBM, Armonk, NY, USA) Statistical Package for the Social Sciences (spss) version 23.0. Descriptive statistics were used to summarise the characteristics of the participants. To evaluate the LIQSS, a principal component analysis (PCA) was performed with an oblique rotation (direct oblimin) on all items. The Kaiser–Meyer–Olkin measure was used to verify the sampling adequacy, and Bartlett's test of sphericity was used to test whether the correlations between items were sufficiently large for a PCA (Field 2009). A scree plot inspection was used to aid decision on the number of components, after which a PCA was performed with a forced three‐component extraction. Based on the PCA solution, three sub‐scales were computed by computing the unweighted sum of the items with a loading of >0.40 on one component. Cronbach's *α* was calculated to assess the reliability (internal consistency) of each of the sub‐scales.

## Results

In the PCA, the Kaiser–Meyer–Olkin measure of sampling adequacy was 0.86, which indicates that a PCA is suitable for the data (Field 2009). Bartlett's test of sphericity, χ^2^ (496) = 2459.84, *P* < 0.001, showed that the correlations between items were sufficiently large for a PCA. The initial results of the PCA showed that seven components had eigenvalues over Kaiser's criterion 1, and in combination, they explained 65.66% of the variance. Based on the scree plot, there were three to five components that could be derived from the LIQSS. Further inspection of the item loadings on the components revealed that three components fitted the data best, using the guideline that an item loading should be >0.30 on one component, with a minimal difference in loading of 0.20 on the other components. Therefore, a PCA was performed with a forced three‐component extraction (Appendix A1). The items that have a high loading (i.e. >0.40) on the same component (Table 2) suggest that component 1 represents communication, making agreements and coordinating on something with others (e.g. ‘Together with my team, I make clear agreements about the way to guide our clients’ and ‘I keep all those involved up to date about the progress the client has made’); component 2 corresponds to staff's positive approach and their ability to provide tailored support (e.g. ‘I compliment clients while they are learning something, as well as afterwards’ and ‘While clients are learning, I build on what they already know and are able to do’); and component 3 concerns supporting independent performance of clients (e.g. ‘I let clients carry out tasks that they can do themselves’ and ‘If clients do not know how to proceed, I ask (mediating) questions, so they come up with the solution themselves’). Ten items were removed from the questionnaire because they did not load highly (i.e. >0.40) on one component and did not correspond to any of the components or because the difference in loadings between two or three components was smaller than 0.20. The final version of the LIQSS consisted of 22 items, which had factor loadings between 0.44 and 0.91 on their corresponding component and a minimal difference in loading to the other factors of 0.20 (Table 2). The PCA of these 22 items with the three components extracted showed that together they explained 55.31% of the variance. The intercomponent correlation coefficients were small to medium (between 0.20 and 0.36). In terms of reliability, all sub‐scales (derived from the components) were found to be internally consistent (Cronbach's *α* was 0.92, 0.79 and 0.76, respectively). The correlation coefficients between the sub‐scales were also small to medium (between 0.29 and 0.44).

**Table 2:** PCA results for the LIQSS with the final 22 items included (*n* = 142)

		Pattern matrix factor loadings			Communalities (variance accounted for per variable)
		Component 1: communication, agreements and coordination	Component 2: positive encouragement and tailoring	Component 3: supporting independent performance	
Mean component score (SD)		4.94 (1.19)	5.94 (0.71)	5.81 (0.62)	
Cronbach's *α* (internal consistency)		0.92	0.79	0.76	
Item					
1	I keep my team members up to date about the learning goals of clients and how they will be worked on (32)	**0.91**	−0.01	−0.20	0.74
2	I make clear agreements with all those involved (psychologist, personal tutors, legal representatives) about the learning goals and how they will be worked on (31)	**0.90**	0.01	−0.15	0.74
3	I keep all those involved up to date about the progress the client has made (25)	**0.83**	−0.02	−0.04	0.65
4	I make agreements with clients about how they could achieve their learning goals (30)	**0.82**	0.01	0.05	0.71
5	Together with my clients, I formulate their learning goals in a ‘SMART’ way (Specific, Measurable, Acceptable, Realistic, Time‐bound) (29)	**0.77**	−0.06	−0.02	0.57
6	I give other people involved (e.g. psychologist, personal tutors, legal representatives) feedback on their way of guiding clients (24)	**0.76**	−0.03	0.08	0.32
7	Together with clients, I evaluate their progress with regard to their learning goals (18)	**0.71**	0.14	0.09	0.63
8	I stimulate all those involved to practise together with clients what they have learned, and to put this into practice (26)	**0.70**	0.20	−0.07	0.55
9	I ask clients to determine their own learning goals (28)	**0.63**	−0.07	0.24	0.54
10	I give my team members feedback on their way of guiding clients (23)	**0.57**	0.04	0.11	0.39
11	Together with my team, I make clear agreements about the way to guide our clients (21)	**0.45**	−0.13	0.23	0.31
12	I motivate and encourage clients while they are learning something (3)	−0.02	**0.88**	−0.12	0.74
13	I compliment clients while they are learning something, as well as afterwards (4)	−0.00	**0.86**	−0.10	0.71
14	While clients are learning, I build on what they already know and are able to do (10)	−0.01	**0.71**	−0.02	0.51
15	While clients are learning, I make use of to their preferred method of learning (11)	0.13	**0.57**	0.34	0.60
16	I express my confidence towards clients about them reaching their learning goals (2)	0.02	**0.53**	0.27	0.43
17	I let clients themselves think about how they should solve or deal with something (8)	−0.15	−0.05	**0.79**	0.54
18	If clients do not know how to proceed, I ask (mediating) questions, so they come up with the solution themselves (15)	0.21	−0.06	**0.75**	0.71
19	I give clients room to make mistakes (16)	−0.04	0.07	**0.69**	0.49
20	I believe that clients should think and do as much as possible themselves (27)	0.07	−0.00	**0.64**	0.45
21	I let clients carry out tasks that they can do themselves (9)	0.03	0.03	**0.45**	0.22
22	I take care that not too much is asked of clients (that they have to do more or try to do more than they can handle) (17)	0.16	0.16	**0.44**	0.33

For each item, the highest loading on a certain component is presented in bold, which corresponds to component they were assigned to. The original item numbers are presented between parentheses.

PCA, principal component analysis; LIQSS, Leiden Independence Questionnaire for Support Staff; SD, standard deviation.

## Discussion

The objective of this study was to develop a questionnaire that assesses the degree to which support staff promote independence in people with ID. The questionnaire had three meaningful, reliable (internally consistent) components of staff behaviours that are important for promoting independence in people with ID. Based on the results of our initial validation, its face validity appears to be strong.

Three distinct and reliable components of staff behaviour regarding promoting independence were identified with the LIQSS. The first component, or sub‐scale, of the LIQSS was termed ‘communication, agreements and coordination’ and concerned communicating and coordinating on something with others and making agreements about the way to guide people with ID towards their learning goals. Involving other people does not only mean the person with ID or other staff members. It is also important to include the social support network when promoting independence (Hale *et al*. 2011; Young *et al*. 2012; Sandjojo *et al*. in press). The second component, ‘positive encouragement and tailoring’, pertained to staff's behaviour towards people with ID during the learning process. These behaviours concern positive encouragement and adapting the provided support to the existing knowledge, skills and preferred way of learning of an individual. The importance of tailoring self‐management support to individuals' needs has been proposed in previous studies as well (Hale *et al*. 2011; Young *et al*. 2012; Evers *et al*. 2014; Petner‐Arrey and Copeland 2015; Kuijken *et al*. 2016). The third component, ‘supporting independent performance’, related to supporting independent performance by letting people with ID handle things themselves as much as possible, as this could benefit the level of independence of people with ID (Sigafoos *et al*. 2005). The structure and the domains that we found based on the results of the PCA largely match the domains that were assumed to underlie the structure of the questionnaire, based on the list of performance objectives we discerned before designing the questionnaire. Our initial domains of ‘setting goals’ and ‘coordinating with others’ can be clustered into the first component ‘communication, agreements and coordination’. The initial domain of ‘motivating people with ID’ corresponds with the second component, ‘positive encouragement and tailoring’. The third component, ‘supporting independent performance’, is almost similar to our initial domain ‘supporting the learning process’.

There are some limitations to this study. First, we used self‐reports as an outcome measure, which could have led to subjective and socially desirable answers. Staff might not have enough self‐reflection into their own behaviour towards people with ID and might overestimate the degree to which they are promoting the level of independence of their clients. The relatively high scores on the LIQSS, especially on components 2 and 3 (4.9, 5.9 and 5.8, respectively), seem to support this. Further fine tuning could therefore be considered, for example, by including a ‘social desirability scale’ that can detect and control for responses that may be influenced by social desirability (van de Mortel 2008) or by assessing staff responses to vignette scenarios (Wong and Wong 2008). Related to the relatively high scores was the finding that few responses were distributed amongst the lowest three answering categories of the 7‐point scale, showing that these were less discriminative than the higher categories. Perhaps a 5‐point Likert scale fits better, as this was found to yield data of higher quality than a 7‐point scale. Having a higher number of answering categories increases the possibilities of differences in interpretation; therefore, a 5‐point scale may be preferred over a 7‐point scale (Revilla *et al*. 2014). For future research, a larger‐scale study with more participants working within various care organisations is recommended. Furthermore, research is necessary on the final version of the LIQSS with 22 items, to replicate the factor structure found in this study and to examine other aspects of the LIQSS's reliability and validity (e.g. inter‐rater reliability or construct validity), as well as its sensitivity to change.

In this study, we describe and initially validate the first questionnaire that assesses the degree to which support staff promote independence in people with ID. Although the questionnaire could profit from further validation and fine tuning to minimise socially desirable answers (Holtgraves 2004; van de Mortel 2008), it has a high potential and promise for use in both scientific research and clinical practice. For example, studies could use the LIQSS to examine which factors influence staff's behaviour in relation to promoting independence and to evaluate staff trainings that target these types of behaviour. Care organisations could use the LIQSS as an assessment instrument to evaluate staff, for example, for training purposes. An independent coach could observe staff and give feedback with the help of the LIQSS. The LIQSS could also be used as a self‐reflection instrument, to create awareness amongst staff about which behaviours contribute to the promotion of independence in people with ID, thereby possibly contributing to behavioural change. These efforts could all contribute to improvements in the support provided by staff and thereby enhance the lives of people with ID.

## Conflict of Interest

The authors declare that they have no conflicts of interest.

## Source of funding

Funding for the study was provided by Raamwerk and Leiden University. This funding had no influence on the collection, analysis and interpretation of the data; on the writing of the report; or on the decision to submit the article for publication.

 

**Table A1:** PCA results for the LIQSS with all 32 items included

		Pattern matrix factor loadings		
		Component 1: communication, agreements and coordination	Component 2: positive encouragement and tailoring	Component 3: supporting independent performance of clients
Item (with original item number)				
2	I express my confidence towards clients about them reaching their learning goals.	0.02	0.51	−0.22
3	I motivate and encourage clients while they are learning something.	−0.05	0.89	0.17
4	I compliment clients while they are learning something, as well as afterwards.	−0.03	0.86	0.15
8	I let clients themselves think about how they should solve or deal with something.	−0.14	−0.11	−0.77
9	I let clients carry out tasks that they can do themselves.	0.07	0.01	−0.38
10	While clients are learning, I build on what they already know and are able to do.	−0.02	0.71	0.04
11	While clients are learning, I make use of to their preferred method of learning.	0.13	0.57	−0.30
15	If clients do not know how to proceed, I ask (mediating) questions, so they come up with the solution themselves.	0.25	−0.12	−0.74
16	I give clients room to make mistakes.	−0.01	0.01	−0.67
17	I take care that not too much is asked of clients (that they have to do more or try to do more than they can handle).	0.17	0.14	−0.43
18	Together with clients, I evaluate their progress with regard to their learning goals.	0.71	0.16	−0.09
21	Together with my team, I make clear agreements about the way to guide our clients.	0.48	−0.16	−0.26
23	I give my team members feedback on their way of guiding clients.	0.58	0.04	−0.11
24	I give other people involved (e.g. psychologist, personal tutors, legal representatives) feedback on their way of guiding clients.	0.77	−0.03	−0.04
25	I keep all those involved up to date about the progress the client has made.	0.82	−0.01	0.05
26	I stimulate all those involved to practise together with clients what they have learned, and to put this into practice.	0.69	0.20	0.09
27	I believe that clients should think and do as much as possible themselves.	0.10	−0.09	−0.61
28	I ask clients to determine their own learning goals.	0.64	−0.08	−0.20
29	Together with my clients, I formulate their learning goals in a ‘SMART’ way (Specific, Measurable, Acceptable, Realistic, Time‐bound).	0.80	−0.07	0.08
30	I make agreements with clients about how they could achieve their learning goals.	0.83	0.01	−0.01
31	I make clear agreements with all those involved (psychologist, personal tutors, legal representatives) about the learning goals and how they will be worked on.	0.88	0.04	0.17
32	I keep my team members up to date about the learning goals of clients and how they will be worked on.	0.92	−0.01	0.25
Deleted item				
1	I express my confidence towards clients about their possibilities/abilities (what they are able to do).	−0.02	0.25	−0.35
5	I remain patient and ‘affectively neutral’ (i.e. not angry or irritated) during setbacks and/or in the absence of success.	−0.15	0.10	−0.43
6	After setbacks and/or in the absence of success, I evaluate the situation with clients (for example regarding what went wrong or how it could be/could have been done better).	0.17	0.44	−0.36
7	I give constructive feedback after a setback and/or in the absence of success.	0.04	0.44	−0.45
12	I make sure that clients work towards their goal in small steps.	0.33	0.33	−0.34
13	I explain to clients why they are learning something, so they see the importance of it.	0.36	0.16	−0.51
14	I change my teaching method/approach if clients get stuck.	0.36	0.46	−0.20
19	I tell clients how they should solve or deal with something.	−0.24	−0.08	0.30
20	I stimulate clients to keep practising and keep putting what they have learnt into practice.	0.25	0.30	−0.23
22	I am on the same page as my team, regarding the way to guide our clients.	0.18	−0.02	−0.06

Participants were instructed to carefully read the items and to indicate to what extent these items were been applicable to them in the past 2 weeks on a scale from 1 (*not at all/never*) to 7 (*completely/always*).

PCA, principal component analysis; LIQSS, Leiden Independence Questionnaire for Support Staff.

## References

[jir12574-bib-0001] Aldridge J. (2010) Promoting the independence of people with intellectual disabilities. Learning Disability Practice 13, 31–36.

[jir12574-bib-0002] Bartholomew L. K. , Parcel G. S. , Kok G. , Gottlieb N. H. & Fernández M. E. (2011) Planning Health Promotion Programs: An Intervention Mapping Approach. Jossey‐Bass, San Francisco.

[jir12574-bib-0004] Beadle‐Brown J. , Hutchinson A. & Whelton B. (2012) Person‐centred active support – increasing choice, promoting independence and reducing challenging behaviour. Journal of Applied Research in Intellectual Disabilities 25, 291–307. 22711478 10.1111/j.1468-3148.2011.00666.x

[jir12574-bib-0005] Bond R. J. & Hurst J. (2010) How adults with learning disabilities view living independently. British Journal of Learning Disabilities 38, 286–292.

[jir12574-bib-0006] Browder D. M. & Shapiro E. S. (1985) Applications of self‐management to individuals with severe handicaps: a review. Journal of the Association for Persons with Severe Handicaps 10, 200–208.

[jir12574-bib-0007] Dollar C. A. , Fredrick L. D. , Alberto P. A. & Luke J. K. (2012) Using simultaneous prompting to teach independent living and leisure skills to adults with severe intellectual disabilities. Research in Developmental Disabilities 33, 189–195. 22093664 10.1016/j.ridd.2011.09.001

[jir12574-bib-0008] Dusseljee J. C. E. , Rijken P. M. , Cardol M. , Curfs L. M. G. & Groenewegen P. P. (2011) Participation in daytime activities among people with mild or moderate intellectual disability. Journal of Intellectual Disability Research 55, 4–18. 21029235 10.1111/j.1365-2788.2010.01342.x

[jir12574-bib-0009] Evers A. W. M. , Gieler U. , Hasenbring M. I. & van Middendorp H. (2014) Incorporating biopsychosocial characteristics into personalized healthcare: a clinical approach. Psychotherapy and Psychosomatics 83, 148–157. 24732828 10.1159/000358309

[jir12574-bib-0010] Felce D. , Bowley C. , Baxter H. , Jones E. , Lowe K. & Emerson E. (2000) The effectiveness of staff support: evaluating active support training using a conditional probability approach. Research in Developmental Disabilities 21, 243–255. 10983781 10.1016/s0891-4222(00)00040-8

[jir12574-bib-0011] Ferretti R. P. , Cavalier A. R. , Murphy M. J. & Murphy R. (1993) The self‐management of skills by persons with mental retardation. Research in Developmental Disabilities 14, 189–205. 8316682 10.1016/0891-4222(93)90030-n

[jir12574-bib-0012] Field A. (2009) Discovering Statistics Using SPSS. SAGE Publications, London.

[jir12574-bib-0013] French D. P. , Cooke R. , Mclean N. , Williams M. & Sutton S. (2007) What do people think about when they answer theory of planned behaviour questionnaires? A ‘think aloud’ study. Journal of Health Psychology 12, 672–687. 17584818 10.1177/1359105307078174

[jir12574-bib-0014] Haigh A. , Lee D. , Shaw C. , Hawthorne M. , Chamberlain S. , Newman D. W. *et al* (2013) What things make people with a learning disability happy and satisfied with their lives: an inclusive research project. Journal of Applied Research in Intellectual Disabilities 26, 26–33. 23255376 10.1111/jar.12012

[jir12574-bib-0015] Hale L. A. , Trip H. T. , Whitehead L. & Conder J. (2011) Self‐management abilities of diabetes in people with an intellectual disability living in New Zealand. Journal of Policy and Practice in Intellectual Disabilities 8, 223–230.

[jir12574-bib-0016] Harchik A. E. , Sherman J. A. & Sheldon J. B. (1992) The use of self‐managment procedures by people with developmental disabilities: a brief review. Research in Developmental Disabilities 13, 211–227. 1626080 10.1016/0891-4222(92)90026-3

[jir12574-bib-0017] Hastings R. P. (2010) Support staff working in intellectual disability services: the importance of relationships and positive experiences. Journal of Intellectual and Developmental Disability 35, 207–210. 20809882 10.3109/13668250.2010.492710

[jir12574-bib-0018] Holtgraves T. (2004) Social desirability and self‐reports: testing models of socially desirable responding. Personality and Social Psychology Bulletin 30, 161–172. 15030631 10.1177/0146167203259930

[jir12574-bib-0019] Kuijken N. M. J. , Naaldenberg J. , Nijhuis‐van der Sanden M. W. & Van Schrojenstein‐Lantman de Valk H. M. J. (2016) Healthy living according to adults with intellectual disabilities: towards tailoring health promotion initiatives. Journal of Intellectual Disability Research 60, 228–241. 26625732 10.1111/jir.12243

[jir12574-bib-0020] Van Laarhoven T. & Van Laarhoven‐Myers T. (2006) Comparison of three video‐based instructional procedures for teaching daily living skills to persons with developmental disabilities. Education and Training in Developmental Disabilities 41, 365–381.

[jir12574-bib-0021] Netherlands Institute for Social Research (2014) Zorg Beter Begrepen. Verklaringen voor de groeiende vraag naar zorg voor mensen met een verstandelijke beperking. Sociaal en Cultureel Planbureau, Den Haag.

[jir12574-bib-0022] Petner‐Arrey J. & Copeland S. R. (2015) ‘You have to care.’ Perceptions of promoting autonomy in support settings for adults with intellectual disability. British Journal of Learning Disabilities 43, 38–48.

[jir12574-bib-0023] Revilla M. A. , Saris W. E. & Krosnick J. A. (2014) Choosing the number of categories in agree–disagree scales. Sociological Methods & Research 43, 73–97.

[jir12574-bib-0024] Rose N. , Rose J. & Kent S. (2012) Staff training in intellectual disability services: a review of the literature and implications for mental health services provided to individuals with intellectual disability. International Journal of Developmental Disabilities 58, 24–39.

[jir12574-bib-0025] Sandjojo, J. , Eltringham, E. G. , Gebhardt, W. A. , Zedlitz, A. M. E. E. , Hoekman, J. , den Haan, J. A. *et al* (in preparation) Interventions for promoting self‐management in daily life for people with ID: a systematic review of studies' effectiveness and applied behavioural change techniques.

[jir12574-bib-0026] Sandjojo J. , Gebhardt W. A. , Zedlitz A. M. E. E. , Hoekman J. , den Haan J. A. & Evers A. W. M. (in press) Promoting independence of people with intellectual disabilities: a focus group study. Perspectives from people with intellectual disabilities, legal representatives, and support staff. Journal of Policy and Practice in Intellectual Disability, 1–16.

[jir12574-bib-0027] Sandjojo J. , Zedlitz A. M. E. E. , Gebhardt W. A. , Hoekman J. , Dusseldorp E. , den Haan J. A. *et al* (2018) Training staff to promote self‐management in people with intellectual disabilities. Journal of Applied Research in Intellectual Disabilities 31, 840–850. 29479785 10.1111/jar.12440

[jir12574-bib-0028] Schalock R. L. (2004) The concept of quality of life: what we know and do not know. Journal of Intellectual Disability Research 48, 203–216. 15025663 10.1111/j.1365-2788.2003.00558.x

[jir12574-bib-0029] Sexton E. , O'Donovan M. A. , Mulryan N. , McCallion P. & McCarron M. (2016) Whose quality of life? A comparison of measures of self‐determination and emotional wellbeing in research with older adults with and without intellectual disability. Journal of Intellectual and Developmental Disability 41, 324–337.

[jir12574-bib-0030] Shogren K. A. , Wehmeyer M. L. , Palmer S. B. , Forber‐Pratt A. J. , Little T. J. & Lopez S. (2015) Causal agency theory: reconceptualizing a functional model of self‐determination. Education and Training in Autism and Developmental Disabilities 50, 251–263.

[jir12574-bib-0031] Sigafoos J. , O'Reilly M. , Cannella H. , Upadhyaya M. , Edrisinha C. , Lancioni G. E. *et al* (2005) Computer‐presented video prompting for teaching microwave oven use to three adults with developmental disabilities. Journal of Behavioral Education 14, 189–201.

[jir12574-bib-0032] Smith K. A. , Shepley S. B. , Alexander J. L. & Ayres K. M. (2015) The independent use of self‐instructions for the acquisition of untrained multi‐step tasks for individuals with an intellectual disability: a review of the literature. Research in Developmental Disabilities 40, 19–30. 25710296 10.1016/j.ridd.2015.01.010

[jir12574-bib-0033] Spriggs A. D. , Mims P. J. , van Dijk W. & Knight V. F. (2017) Examination of the evidence base for using visual activity schedules with students with intellectual disability. Journal of Special Education 51, 14–26.

[jir12574-bib-0035] Totsika V. , Toogood S. , Hastings R. P. & Nash S. (2008) Interactive training for active support: perspectives from staff. Journal of Intellectual and Developmental Disability 33, 225–238. 18752095 10.1080/13668250802283348

[jir12574-bib-0036] United Nations (2006) Convention of the rights of persons with persons with disabilities. Available at: https://www.un.org/development/desa/disabilities/convention‐on‐the‐rights‐of‐persons‐with‐disabilities.html. 10.1515/9783110208856.203 18348362

[jir12574-bib-0037] Van de Mortel T. F. (2008) Faking it: social desirability response bias in self‐report research. Australian Journal of Advanced Nursing 25, 40–48.

[jir12574-bib-0038] Vilaseca R. , Gràcia M. , Beltran F. S. , Dalmau M. , Alomar E. , Adam‐Alcocer A. L. *et al* (2017) Needs and supports of people with intellectual disability and their families in Catalonia. Journal of Applied Research in Intellectual Disabilities 30, 33–46. 26423573 10.1111/jar.12215

[jir12574-bib-0040] Wehmeyer M. L. , Kelchner K. & Richards S. (1996) Essential characteristics of self determined behavior of individuals with mental retardation. American Journal on Mental Retardation 100, 632–642. 8735576

[jir12574-bib-0041] Wong P. K. S. & Wong D. F. K. (2008) Enhancing staff attitudes, knowledge and skills in supporting the self‐determination of adults with intellectual disability in residential settings in Hong Kong: a pretest–posttest comparison group design. Journal of Intellectual Disability Research 52, 230–243. 18261022 10.1111/j.1365-2788.2007.01014.x

[jir12574-bib-0042] Young A. F. , Naji S. & Kroll T. (2012) Support for self‐management of cardiovascular disease by people with learning disabilities. Family Practice 29, 467–475. 22071441 10.1093/fampra/cmr106

